# A role for multiple chimeric antigen receptor-expressing leukocytes in antigen-specific responses to cancer

**DOI:** 10.18632/oncotarget.9149

**Published:** 2016-05-03

**Authors:** Carmen S.M. Yong, Liza B. John, Christel Devaud, Miles H. Prince, Ricky W. Johnstone, Joseph A. Trapani, Phillip K. Darcy, Michael H. Kershaw

**Affiliations:** ^1^ Sir Peter MacCallum Department of Oncology, University of Melbourne, Parkville, Victoria, Australia; ^2^ Institut de Recherche en Santé Digestive, Université de Toulouse, INPT, INRA, INSERM UMR1220, UPS, France; ^3^ Department of Immunology, Monash University, Prahran Victoria, Australia

**Keywords:** T cells, macrophages, NK cells, chimeric antigen receptor, transgenic mouse

## Abstract

While adoptive immunotherapy using chimeric antigen receptor (CAR)-modified T cells can induce remission of some tumors, the role of other CAR-modified leukocytes is not well characterized. In this study, we characterize the function of leukocytes including natural killer (NK) cells, macrophages and CAR T cells from transgenic mice expressing a CAR under the control of the pan-hematopoietic promoter, *vav*, and determine the ability of these mice to respond to ERB expressing tumors. We demonstrate the anti-tumor functions of leukocytes, including antigen specific cytotoxicity and cytokine secretion. The adoptive transfer of CAR T cells provided a greater survival advantage in the E0771ERB tumor model than their wildtype (WT) counterparts. In addition, CAR NK cells and CAR T cells also mediated increased survival in the RMAERB tumor model. When challenged with Her2 expressing tumors, F38 mice were shown to mount an effective immune response, resulting in tumor rejection and long-term survival. This was shown to be predominantly dependent on both CD8^+^ T cells and NK cells. However, macrophages and CD4^+^ T cells were also shown to contribute to this response. Overall, this study highlights the use of the *vav*-CAR mouse model as a unique tool to determine the anti-tumor function of various immune subsets, either alone or when acting alongside CAR T cells in adoptive immunotherapy.

## INTRODUCTION

Adoptive immunotherapy has demonstrated great clinical success as a form of treatment for cancer. In particular, the adoptive transfer of cytotoxic lymphocytes (CTLs) genetically modified to express a chimeric antigen receptor (CAR) specific for a tumor associated antigen (TAA) has shown great promise, with the most recent clinical success in blood cancers such as Chronic Lymphocytic Leukemia (CLL) and Acute Lymphoblastic Leukemia (ALL) [1–5]. However, the use of adoptively transferred CAR T cells into solid cancers has been met with numerous hurdles, and subsequently has struggled to gain similar success [6, 7]. In leukemic or lymphoma models of disease, adoptive transfer of genetically modified T cells come into contact with the tumor cells almost immediately, reducing the need to localize and persist at the tumor site. In contrast, solid tumors present numerous challenges; with issues arising involving the persistence and survival of transferred T cells, the inability of T cells to penetrate the tumor mass and maintaining their effector cell function within an immunosuppressive tumor microenvironment [8–10]. The ability of CAR T cells to overcome these multiple factors alone has proven to be inadequate. However, these issues may be resolved by using alternate types of CAR-expressing leukocytes, either alone or in combination.

Evidence suggests that concurrent transfer of other immune subsets may overcome some of the issues in tackling solid tumors using adoptive immunotherapy, either through directly increasing the effector function of CTLs or by modification of the surrounding tumor microenvironment into an anti-tumoral environment. Multiple studies have demonstrated an enhanced anti-tumor effect when both CD4^+^ and CD8^+^ T cells were adoptively transferred, compared to CD8^+^ T cells alone [11–14]. Furthermore, the adoptive transfer of irradiated macrophages has been shown to aid in the localization and activity of adoptively transferred T cells through re-polarisation of the tumor microenvironment, facilitating a more receptive environment for T cell function [15]. While a handful of studies have hinted at the potential of CAR modified monocytes, very few studies have attempted to fully understand or demonstrate their ability to function via a CAR [16, 17]. Furthermore, whilst CAR expressing CD8^+^ T cells, and to a much lesser extent NK cells, have been studied in detail, there has been little work into the role and function of other immune subsets expressing a CAR [18–25].

In order to study the role of various immune subsets when genetically modified with a CAR, we generated a transgenic mouse model in which all immune cells express the same chimeric antigen receptor recognizing the same tumor antigen. To ensure the expression of the CAR was limited specifically to immune cells, we utilized the *vav* promoter, a protein whose expression is restricted to cells of the hematopoietic lineage, to drive the expression of the chimeric antigen receptor. Previous transgenic models utilizing the *vav* promoter have demonstrated its capability in driving transgene expression in all immune subsets regardless of lineage or maturation state [26–28]. The chimeric antigen receptor used in our studies is comprised of T cell intracellular signaling domains, derived from CD3ζ and CD28, linked to an extracellular single chain variable fragment specific for the tumor antigen, ErbB2 (Her2). Using this novel mouse model, we recently validated the ability of the *vav* promoter to drive the expression of a chimeric antigen receptor throughout multiple immune subsets [29]. We generated two different strains of the *vav*-CAR mice, each with varying immune compositions and levels of receptor expression. F9 mice harboured almost 270 copies of the transgene, had high levels of receptor expression however were distinctly reduced in the number of B and T lymphocytes present in the thymus, lymph nodes and spleen. F38 mice were normal in immune composition but had a lower level of receptor expression, with only 7 copies of the *vav*-CAR transgene present in these mice.

In the present study, we report that the presence of the CAR enables recognition and subsequent multifaceted effector functions in both NK and T cells, and also suggests an important antigen-specific role for macrophages and CD4^+^ T cells in tumor rejection. This mouse model may provide a deeper understanding of the capabilities of other CAR bearing immune subsets in the context of adoptive immunotherapy.

## RESULTS

We have previously shown the *vav* promoter was proficient in driving the expression of the CAR against the human Her2 antigen in a range of hematopoietic cells, of both myeloid and lymphoid origin in two transgenic mouse models [29]. Immune characterization and phenotypic studies of F9 mice revealed that the high copy number of transgenes present had altered the T cell development of these mice, resulting in an abnormal immune composition with a severe reduction in the proportion of lymphocytes. The immune composition of F38 mice was comparable to WT mice, with high levels of CAR expression observed on all immune subsets with the exception of B cells. Having established the expression of the receptor on multiple immune subsets, we next explored the functional relevance of the CAR in these immune cells.

### Transgenic CAR T cells display antigen specific function

**Figure 1:**
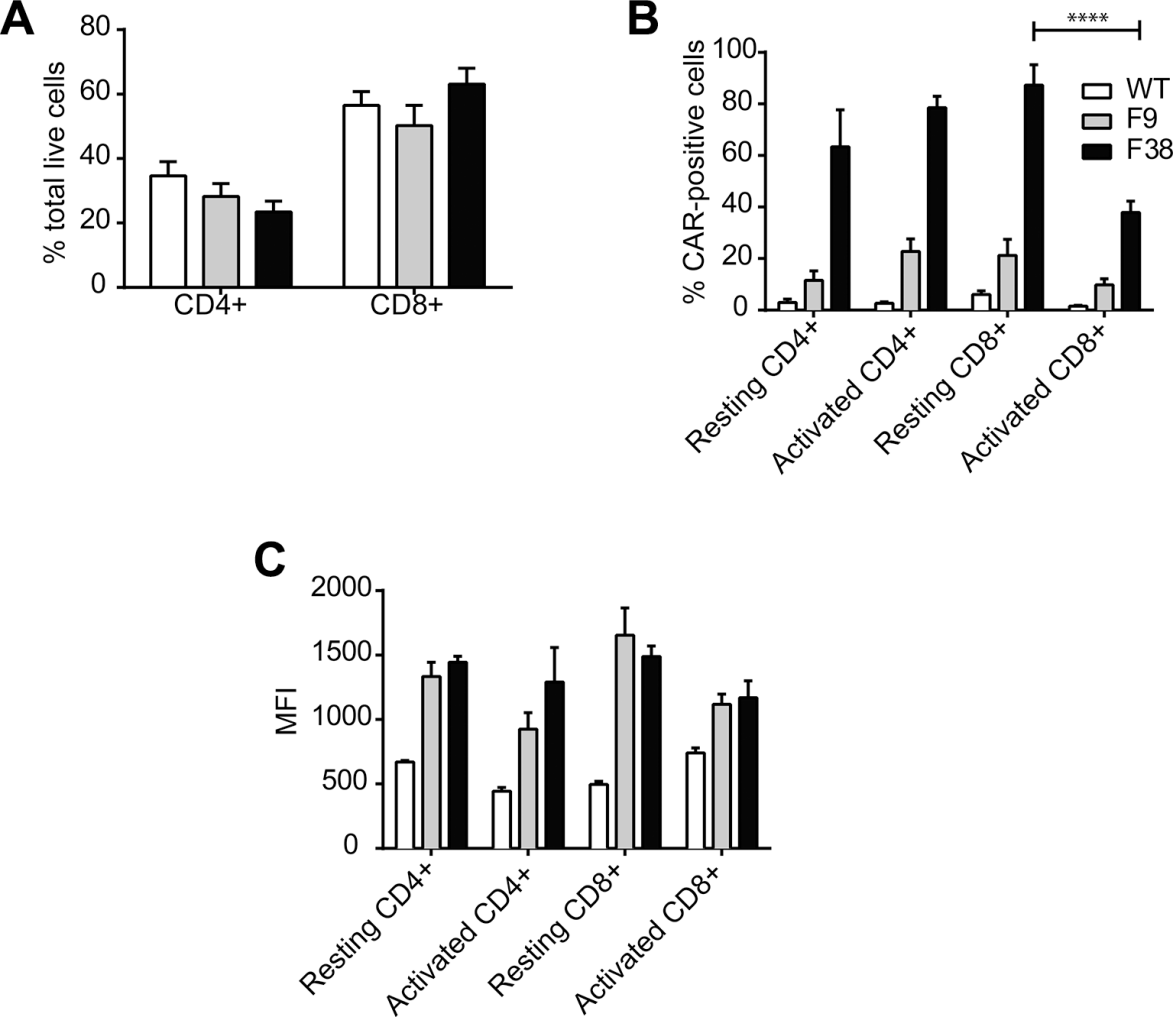
CAR expression in naïve and activated T cells Splenocytes from WT, F9 and F38 mice were harvested and activated overnight with α-CD3ε and α-CD28 in the presence of rhIL-2 and mIL-7 and cultured *in vitro* for 5 days. Activated T cells were then stained for FACS with the following markers; CD3, TCRb, CD4, CD8, α-c-myc tag-A488 and IgG2a-A488. (**A**) The proportion of CD4^+^ and CD8^+^ T cells, (**B**) CAR^+^ T lymphocytes and (**C**) the mean fluorescence intensity (MFI) of CAR expression on gated lymphocytes was analysed. The MFI of cells from wild type mice represents the negative control level of fluorescence. Data represents mean ± standard error of the mean (SEM) of 4 (resting) or 10 (activated) independent experiments ****p < 0.0001.

The majority of adoptive transfer studies have utilized CD8^+^ T lymphocytes, exploiting their ability to secrete high levels of cytokine and mediate immediate cytolytic killing upon antigen recognition. We had previously validated the level of CAR expression on resting CD8^+^ T lymphocytes, however it was not clear whether CAR expression differed post activation. As adoptive transfer experiments often require long periods of *in vitro* expansion, stable CAR expression during activation and proliferation remains an integral factor for these studies. We observed similar proportions of CD4^+^ and CD8^+^ T cells from 5-day *in vitro* cultured (activated) *vav*-CAR and WT mice splenocytes activated with a combination of anti-CD3/28 and recombinant cytokines (Figure 1A). However, while the proportion and level of CAR expression (as measured by mean fluorescence intensity (MFI)) on CD4^+^ T lymphocytes remained stable over the 5 day culture, we observed a decrease in both proportion and level of CAR expression on CD8^+^ T lymphocytes stimulated the same way (Figure 1B–1C).

**Figure 2:**
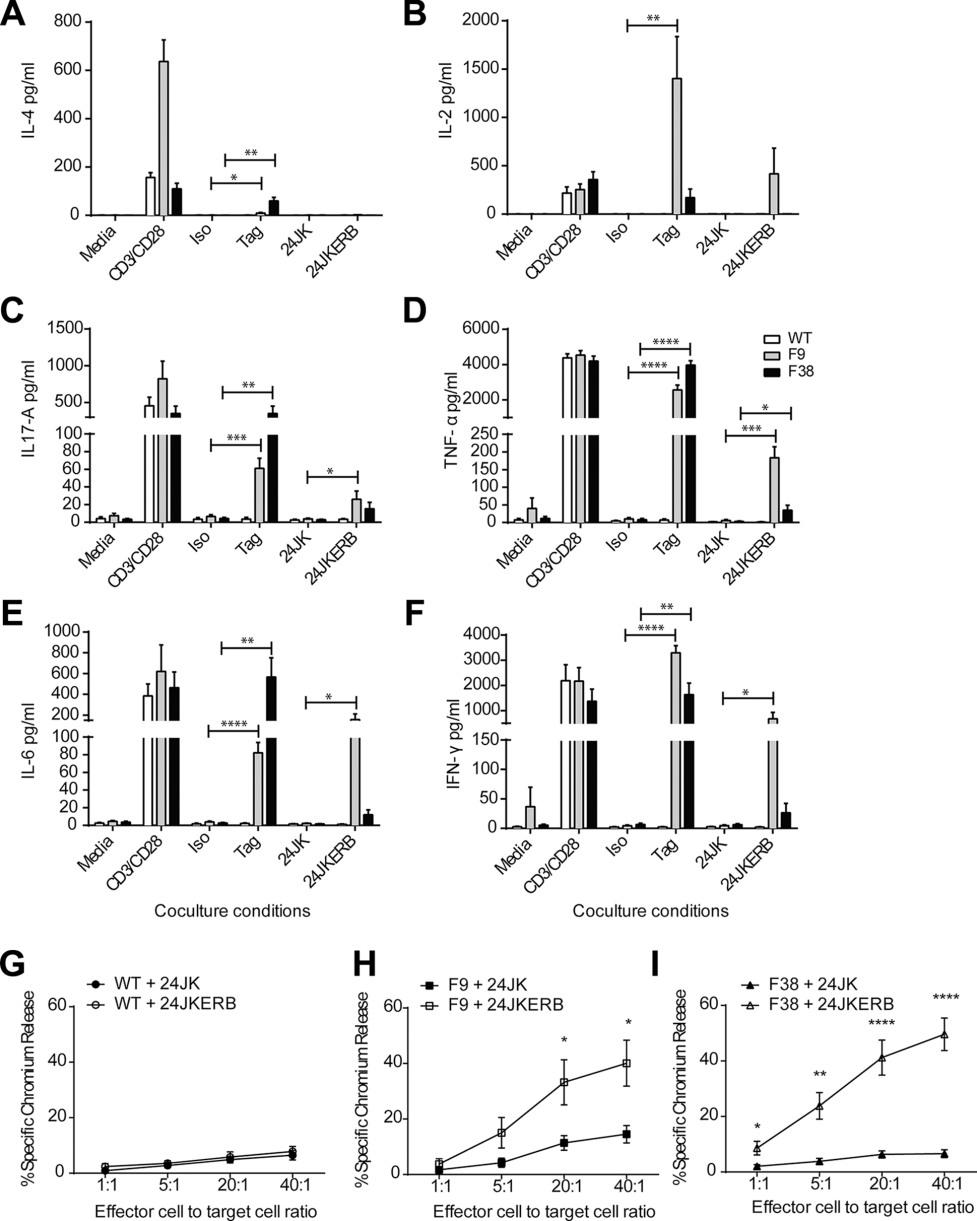
Antigen specific function from CAR T cells (**A**–**F**) *In vitro* activated T cells were co-cultured overnight in the presence of media, immobilized anti-CD3ε and anti-CD28, agonistic c-myc tag or isotype IgG2a and tumor targets 24JK and 24JKERB. Supernatant was harvested the next day and analysed for cytokine secretion using cytometric bead array. Data represents mean ± SEM for 4 independent experiments (total of 7–8 mice/strain). (**G**–**I**) *In vitro* activated T cells were co-cultured with ^51^Cr labeled tumor targets 24JK or 24JKERB in a 4-hour killing assay at various effector to target ratios. The level of antigen-specific killing was assessed by the amount of free chromium released into the supernatant after 4 hours. Data represents mean ± SEM for 6 independent experiments (total of 7–8 mice/strain). *p < 0.03, **p < 0.009, ***p < 0.0004, ****p < 0.0001.

Having demonstrated expression of the CAR on these T cells, we then further characterized whether the CAR was functional. The ability of T lymphocytes to recognize and respond to antigen *in vitro* has commonly been assessed through measuring the release of cytokine. *In vitro* activated T lymphocytes were co-cultured overnight with a specific agonistic antibody for the CAR (α-c-myc tag), or with the fibrosarcoma cell line 24JK expressing the ERB antigen (24JKERB). Stimulation through the isotype antibody (IgG2a) or antigen negative targets (24JK) induced neglible levels of cytokine from CAR T cells. However, following stimulation with α-c-myc tag monoclonal antibody or the tumor cells 24JKERB, we detected a range of cytokines, including IL-4, IL-2, IL-17A, TNF-α, IL-6 and IFN-γ from CAR T cells, while no significant response was observed from WT T cells (Figure 2A–2F).

In addition to cytokine production, cytotoxicity is another measure of T cell function. Moreover, the ability to unleash immediate cytotoxic function upon recognition of a target antigen is one of the pivotal characteristics that has driven the success of CAR T cells in adoptive immunotherapy. We sought to assess the ability of CAR T cells to kill in an antigen specific manner in a chromium release assay. WT T cells showed similarly low levels of killing from both the parental and ERB expressing targets (Figure 2G). Both F9 and F38 T cells were capable of mediating antigen specific cytotoxicity against the tumor line 24JKERB while minimal killing was observed against the antigen negative control 24JK (Figure 2H–2I). This result was even observed at a 1:1 ratio for F38 T cells, indicating CAR T cells are capable of mediating a high level of antigen specific cytotoxicity at reduced effector:target ratios

### *In vitro* cytokine and cytotoxicity from CAR NK cells

The use of CAR-bearing NK cells has also shown great promise as a form of therapy, however due to the difficulty in generating sufficient numbers of gene-modified primary murine NK cells, a majority of studies have utilized NK cell lines or human NKs [25, 30–32]. Therefore, the use of primary mouse NK cells and their function when genetically modified with a CAR has not been extensively studied. We had previously established the ability of primary mouse NK cells electroporated to express a CAR, however expression of the CAR was transient in these cells due to the method of genetic modification [22]. One caveat of utilising NK cells for adoptive immunotherapy is the need to overcome inhibitory signals, which may dampen the immune response. It is well established that the killing capability of NK cells relies on a fine balance between the level of activating and inhibitory signals received. Interaction of the major histocompatibility complex class I (MHC Class I) molecule results in triggering of intracellular inhibitory signaling pathways in NK cells and subsequently terminates any effector function [33]. To determine whether the CAR could override the inhibitory signals and induce antigen specific cytokine secretion or cytotoxicity, we added in our experiments the murine lymphoma cell line RMA, which express high levels of MHC Class I.

**Figure 3:**
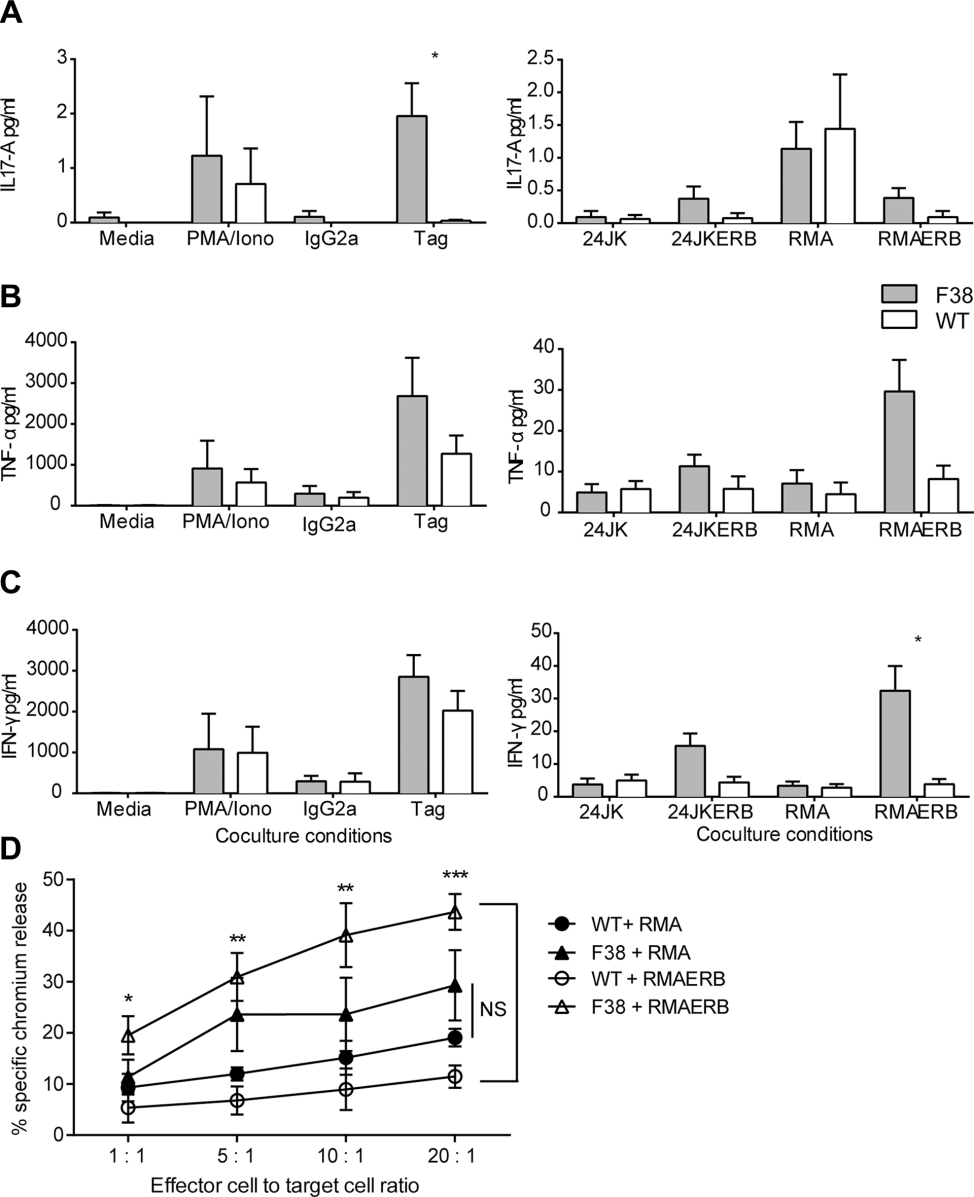
Natural killer cells expressing a CAR can mediate antigen-specific responses (**A**–**C**) *In vitro* activated WT and CAR NK cells were co-cultured overnight in the following conditions; media, PMA/ionomycin, immobilized IgG2a or α-c-myc tag or tumor targets 24JK/24JKERB and RMA/RMAERB at a 1:1 ratio. Supernatant was harvested the following day and assessed for cytokine secretion by CBA. Data represents mean ± SEM for 3 independent experiments (total of 3 mice/strain) *p < 0.03. (**D**) *In vitro* activated WT and CAR NKs were co-cultured with chromium labeled tumor targets RMA/RMAERB at various effector to target ratios and incubated for four hours. The supernatant was then harvested and assessed for the level of free chromium. The percentage of antigen specific cytotoxicity was then determined. Data represents mean ± SEM for 4 independent experiments (total of 4 mice/strain). *p < 0.02, **p < 0.007, ***p = 0.0002.

Given the relatively high level of expression of CAR on NK cells from F38 mice described previously [29], we focused on cells from this mouse strain for our NK cell studies. We detected low levels of multiple cytokines from *in vitro* activated F38 CAR NK cells in response to both 24JKERB and RMAERB target cells, with negligible levels produced against the parental targets (24JK and RMA) or from WT NKs against all targets (Figure 3A–3C). CAR NK cells were observed to secrete a higher level of IL-17A following stimulation with α-c-myc tag antibody, although there was no significant difference when cultured with the antigen expressing tumor targets (Figure 3A). TNF-α secretion was highest when CAR NK cells were co-cultured with either the α-c-myc tag antibody or the RMAERB cell line (Figure 3B), where stimulation with the isotype control antibody (IgG2a) or the parental cell line RMA induced similar levels to WT NK cells. Parallel observations were observed with IFN-γ, with higher levels found in α-c-myc tag stimulated or RMAERB co-cultures (Figure 3C).

We then assessed the cytotoxicity of CAR NK cells in a chromium release assay. Despite similar methods of isolation and activation, we observed an overall higher level of background killing from CAR NK cells against RMA targets compared to WT NKs (Figure 3D). However, while similar levels of cytotoxicity were observed from both WT and CAR NK cells against the parental RMA cell line (Figure 3D, NS at all E:T ratios), a comparison of WT and CAR NK cells co-cultured against RMAERB targets showed highly significant antigen specific cytotoxicity, indicating the ability of CAR NK cells to recognise and perform cytotoxic function through the chimeric antigen receptor (Figure 3D).

**Figure 4:**
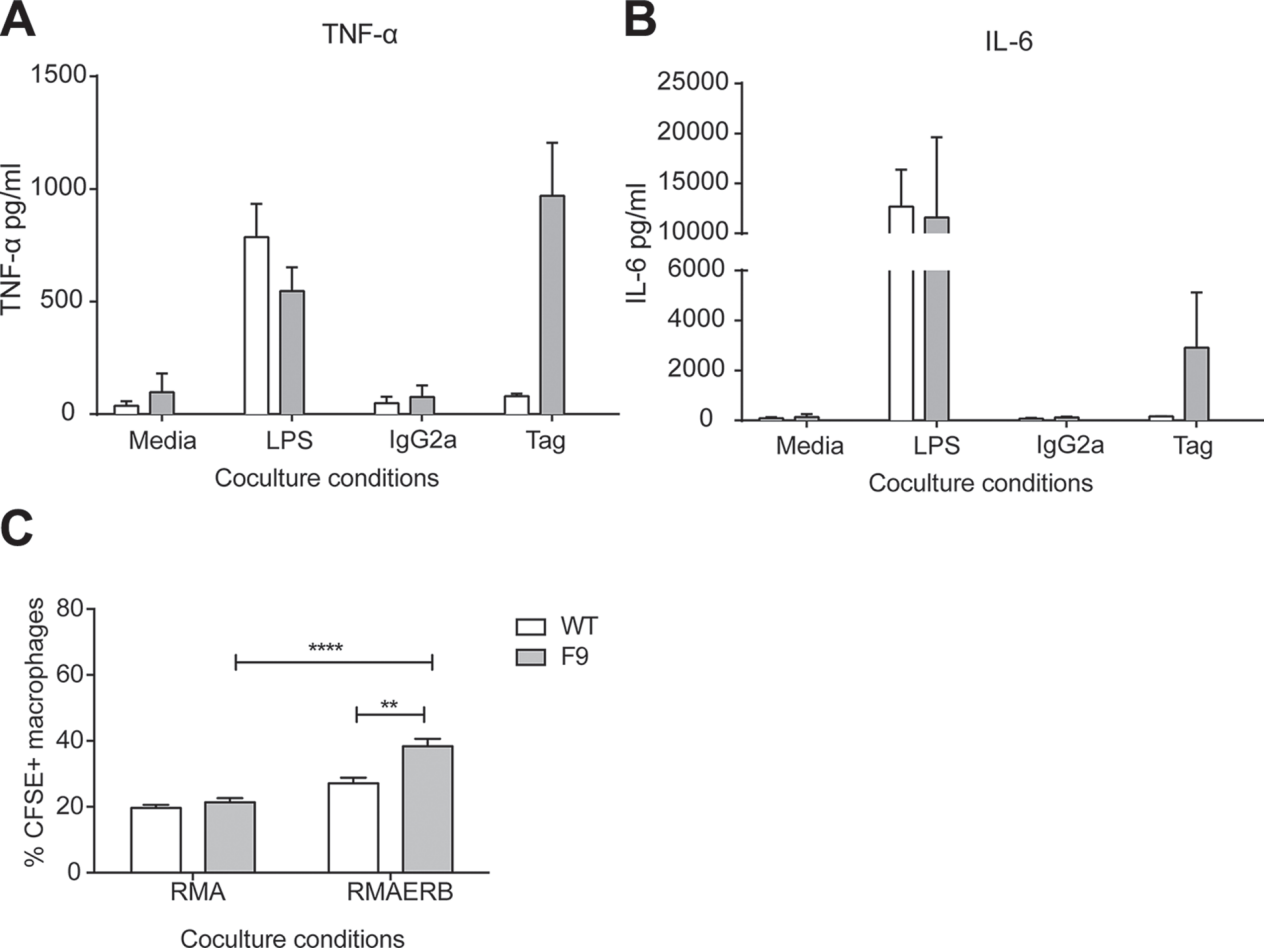
Antigen specific responses from CAR^+^ monocytes (**A**–**B**) Peritoneal exudate cells (PECs) from WT and *vav*-CAR mice were harvested and co-cultured overnight in the following conditions; media, LPS, immobilized IgG2a or α-c-myc tag. Supernatant was harvested the following day and assessed for TNF-α and IL-6 secretion by CBA. Data represents mean ± SEM for 2 independent experiments. (**C**) WT and *vav-*CAR mice were injected intraperitoneally with thioglycollate and PECs harvested 4 days later. Tumor targets were labeled with CFSE and co-cultured with PECs at a 1:1 ratio for an hour at 37°C. Cells were harvested and stained for FACs. Macrophages were gated and analysed for the proportion of CFSE^+^ cells in the population. Data represents mean ± SEM for 3 independent experiments (total of 6 mice/strain) **p < 0.003, ****p < 0.0001.

Since macrophages can also interact directly with microorganisms and diseased cells, we also investigated the antigen specific function of CAR-expressing macrophages *in vitro*. Given our previous findings, we chose to utilise F9 mice in this experiment due to the higher level of CAR expression in macrophages of this strain. Peritoneal exudate cells (PECs) were isolated from naïve WT or F9 mice and assessed for antigen-specific secretion of TNF-α and IL-6 (Figure 4A–4B). We observed higher levels of both cytokines from F9 PECs co-cultured against the α-c-myc tag antibody, with little or no secretion was observed from the IgG2a control or WT PECS. We next assessed if the phagocytic ability of macrophages would be altered by their expression of a CAR. Thioglycollate activated PECs were co-cultured with CFSE-labelled tumor targets and analysed by flow cytometry for the proportion of CFSE^+^ macrophages. A significantly higher proportion of CFSE^+^ macrophages were present in CAR macrophages cultured with RMAERB compared to culture with RMA (Figure 4C). Although further assessment is necessary before we are able to determine any anti-tumor potential of CAR macrophages, these experiments confirmed that the CAR on this cells is functional and can signal for both phagocytic uptake and cytokine secretion.

### Adoptive transfer of CAR T cells or CAR NK cells enhances survival of tumor bearing mice

**Figure 5:**
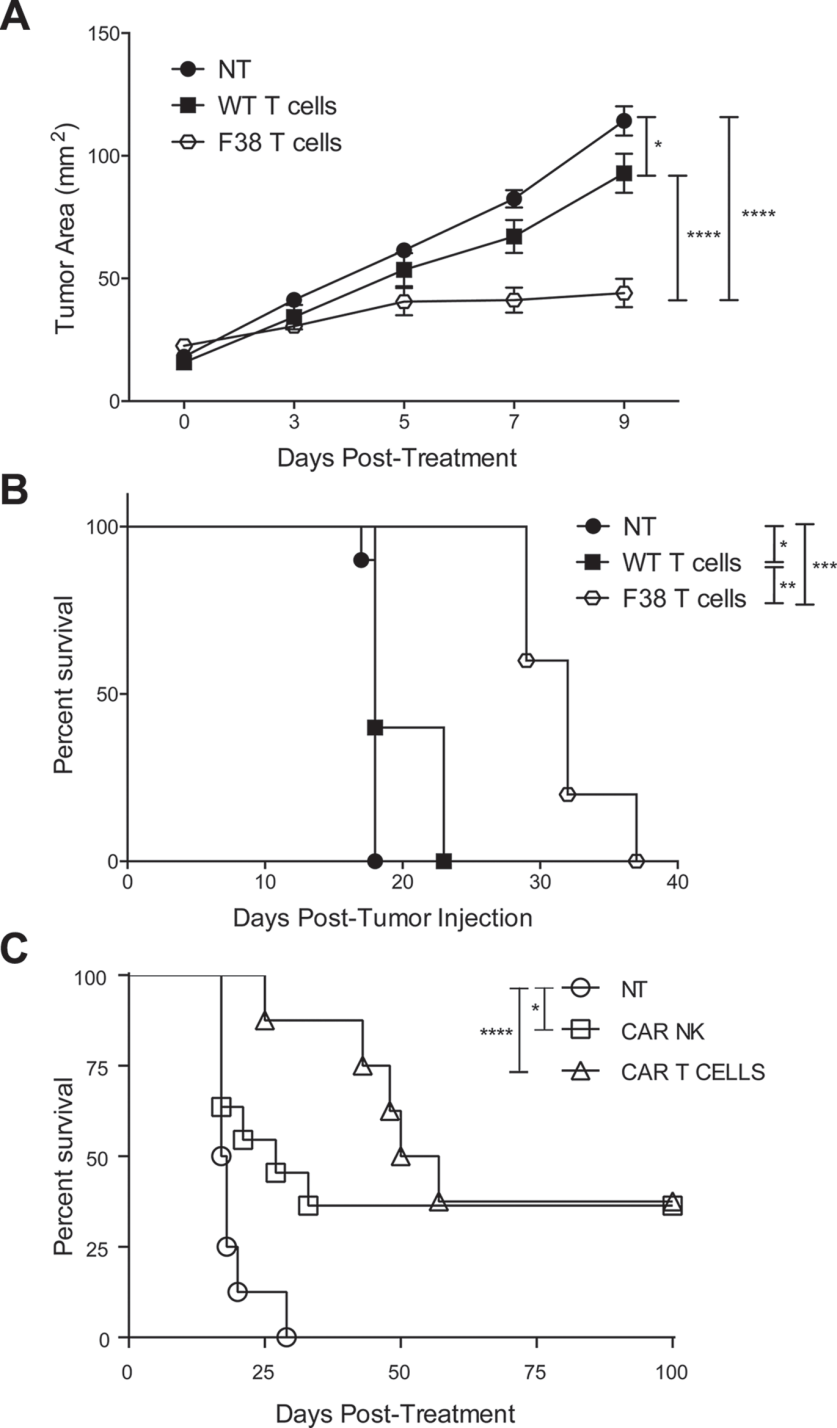
Adoptive transfer of CAR T or CAR NK cells into Her2 tumor bearing mice impedes tumor growth and provides a survival advantage (**A–B**) 1 × 10^5^ E0771ERB cells were injected subcutaneously into Her2 transgenic mice. On day 7 post tumor inoculation, mice were sublethally irradiated with 5 Gy irradiation. *In vitro* activated T cells were adoptively transferred intravenously on days 7, 8 and 14 post tumor inoculation. Exogenous rhIL-2 was administered on the day of day 7 transfer and thereafter twice daily for 4 days post T cell transfer. Data represents one experiment, 5 mice per treated group, 10 mice for non-treated. *p < 0.05, **p < 0.004, ***p < 0.0005, ****p < 0.0001. (**C**) 1 × 10^6^ RMAERB cells were injected into the peritoneum of WT mice. Adoptive transfer of 5 × 10^6^ *in vitro* activated CAR NK cells or CAR T cells were delivered into the peritoneum on the same day. Non-treated mice served as a control. Mice were monitored for signs of sickness or an increase in stomach diameter and sacrificed accordingly. Data represents one experiment, 8-11 mice/group. *p < 0.03, ****p < 0.0001.

The *vav*-CAR mouse was generated as a tool to enable simple and easy isolation of CAR expressing immune subsets without the need for *ex vivo* genetic manipulation. Given the low numbers of T cells present in the F9 strain, we used T cells and NK cells isolated from F38 mice for adoptive transfer experiments. We first assessed the ability of CAR T and CAR NK cells to perform anti-tumor effector responses *in vivo* and whether the effector functions observed *in vitro* would translate in the Her2 mouse model. We found that the adoptive transfer of CAR T cells into E0771ERB subcutaneously tumor-bearing mice significantly inhibited tumor growth compared to mice treated with WT T cells (Figure 5A), and subsequently provided a significant survival advantage (Figure 5B). In a different tumor model, the adoptive transfer of CAR T cells or CAR NK cells into RMAERB tumor bearing mice also significantly increased survival (Figure 5C). This further validated the *in vitro* data and demonstrated that CAR T and CAR NK cells were capable of mediating anti-tumor effects *in vivo*. Future studies incorporating the combination of these two subsets would be important in order to elucidate potential synergistic effects.

### NK cells and T cells are required for tumor rejection in *vav*-CAR mice

In order to expand sufficient cells for therapeutic use, we utilized *in vitro* activated T cells and NK cells in our previous experiments. Whilst we demonstrated that the CAR remained detectable (data not shown) and functional post activation, considerable non-specific cytotoxicity was observed from both WT and CAR NK cells, possibly due to the methods of *in vitro* activation used. Therefore, we next determined whether we could achieve *in vivo* efficacy using naïve effector cells, and furthermore, if the presence of the CAR on multiple immune subsets would enable them to coordinate and mount an effective immune response *in vivo* against a tumor expressing the ERB antigen.

**Figure 6:**
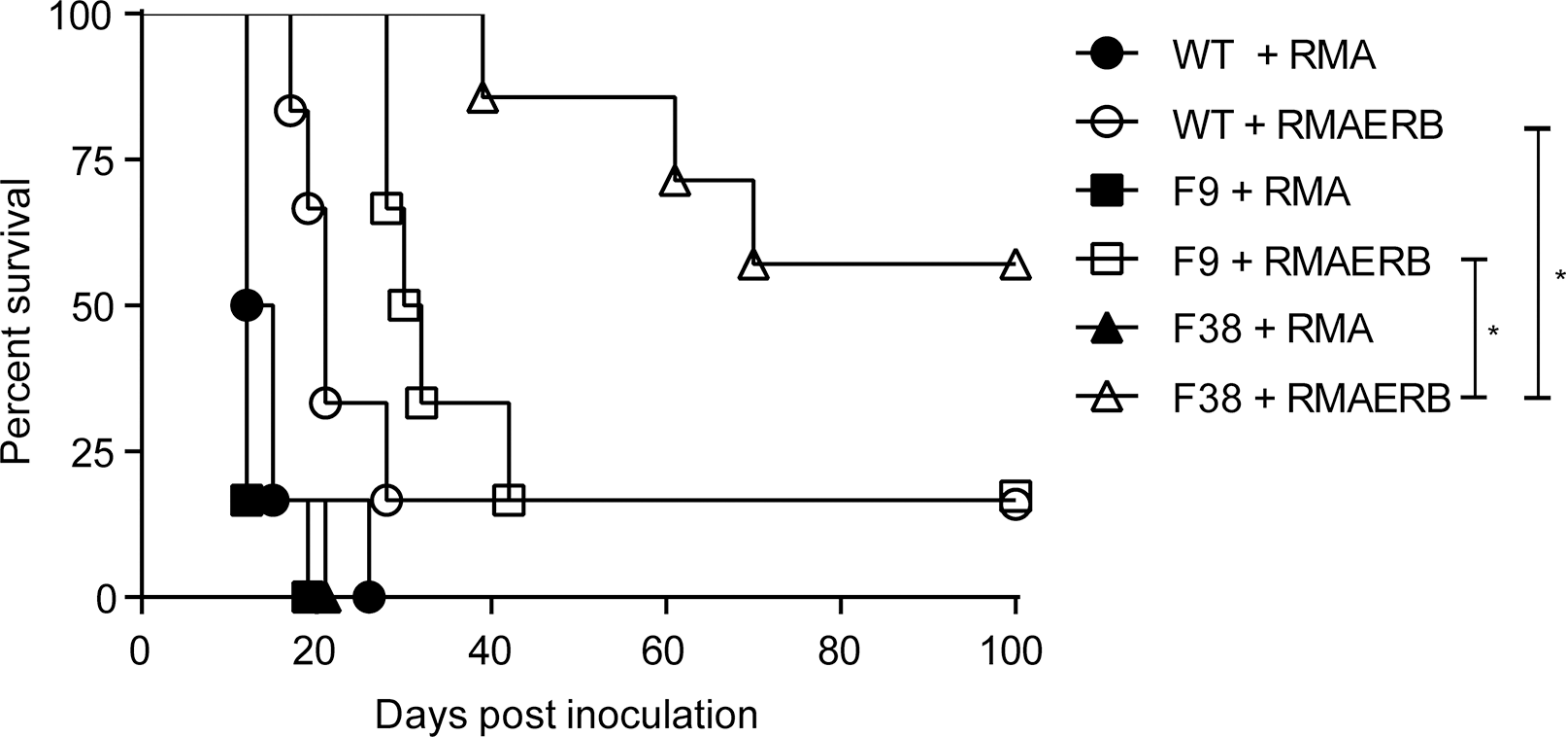
F38 mice can mount an effective immune response against RMAERB tumors *in vivo* Naïve WT, F9 and F38 mice were inoculated with 1 × 10^6^ RMA or RMAERB cells in the peritoneum and monitored for survival. Mice were sacrificed when showing signs of sickness or stomach diameter had increased by 30%, indicative of tumor growth. Data represents one of two independent experiments, 5–7 mice/group per experiment. *p < 0.03.

Naïve WT or *vav-*CAR mice were inoculated in the peritoneum with RMA or RMAERB cells and monitored for survival (Figure 6). CAR expression seemed to have no effect on mice challenged with the RMA cell line, as both WT and vav-CAR mice all succumbed to tumor burden and were sacrificed at a similar timepoint. A significant proportion of F38 mice challenged with RMAERB were able to reject the tumor challenge, resulting in long term survival. In comparison, both WT and F9 mice showed similarly poor survival rates when challenged with RMAERB tumors.

**Figure 7:**
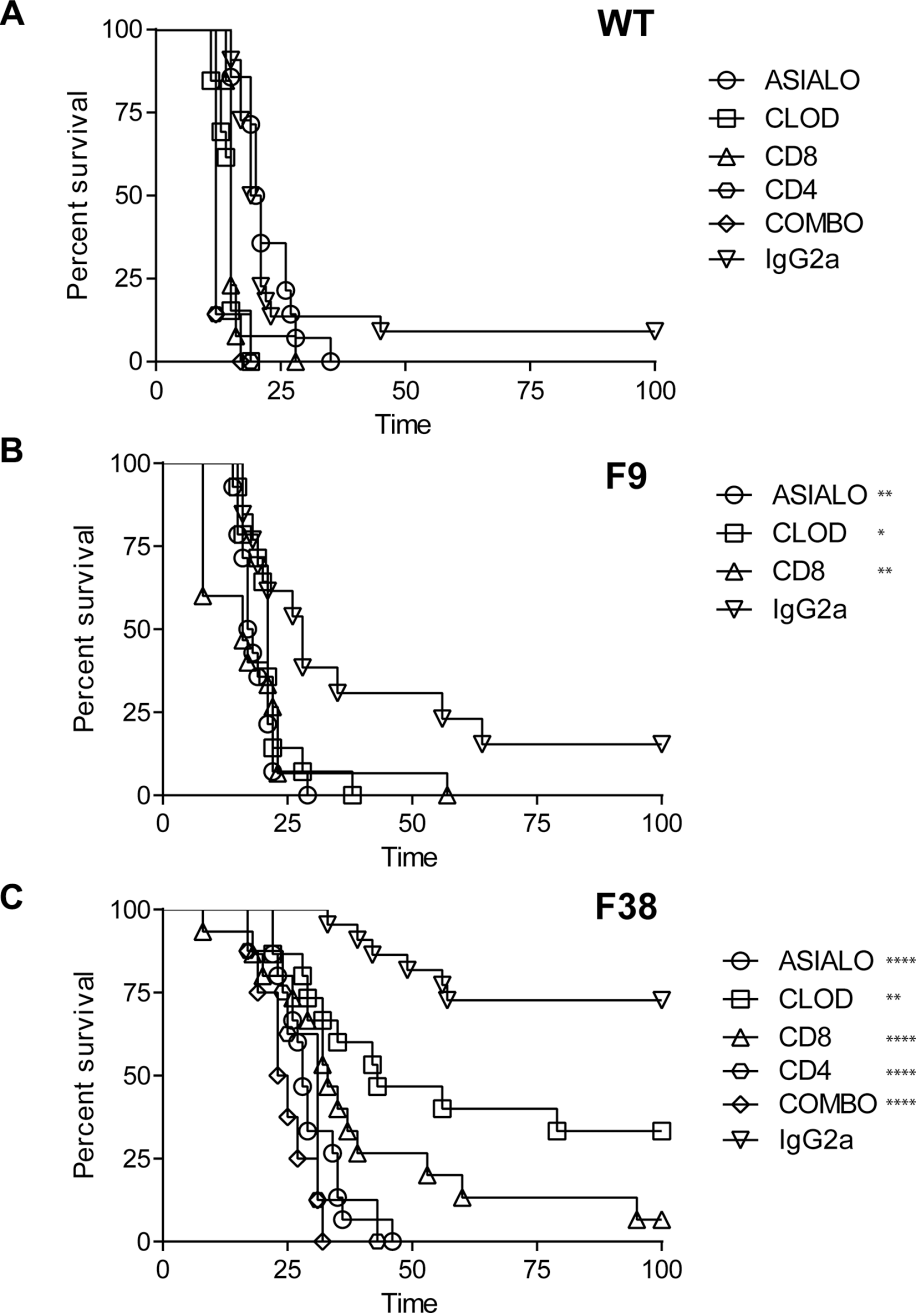
A role for CAR^+^ lymphoid and myeloid cells in tumor rejection in F38 mice (**A**) WT, (**B**) F9 and (**C**) F38 mice were depleted of certain immune subsets with the following reagents; Asialo GM1 (NK cells), Clodronate Liposomes (Clod) (Phagocytes, macrophages), α-CD8 (CD8^+^ T cells), α-CD4 (CD4^+^ T cells), combination (COMBO) (Asialo GM1 and α-CD8) or IgG2a (irrelevant antibody control). Primary depletions were performed 2 days prior and on the day of tumor inoculation. Weekly maintenance depletions were performed for 6–7 weeks post tumor inoculation. Mice were sacrificed when showing signs of sickness or stomach diameter had increased by 30%, indicative of tumor growth. Data represents 2–3 independent experiments, 6–20 mice/group in total. Statistical significance comparing treatment group versus IgG2a treatment supplied on the figure. Comparisons of COMBO vs CD8, *p* = 0.006, COMBO vs ASIALO, *p* = 0.052. *p < 0.03, **p < 0.008, ****p < 0.0001.

To delineate the contribution of each immune subset in this tumor model, we depleted specific immune subsets *in vivo* (with weekly maintenance depletions) prior to RMAERB tumor inoculation. In WT mice, although the depletion of NK cells alone seemed to have no effect on survival, we observed a slight but significant decrease in survival following depletion of macrophages, CD4^+^ and CD8^+^ T cells as well as the combination of both NK cells and CD8^+^ T cells. However, the magnitude of the impact on survival was small. This suggested that endogenous (non-CAR related) immunity could have a minor role in tumor inhibition (Figure 7A). In F9 mice, the depletion of NK cells, macrophages and CD8^+^ T cells was found to reduce the survival advantage compared to irrelevant antibody control (IgG2a) treated mice (Figure 7B), however the median survival time of F9 immunodepleted mice compared to IgG2a-treated was similar. As these mice naturally have significantly lower numbers of T cells, this was result was not surprising. In contrast, in F38 mice, we observed a significant reduction in the survival advantage following immunodepletion with all treatments (Figure 7C). The anti-tumoral contribution of T lymphocytes and NK cells in F38 mice was apparent, with depletions of either subset completely ablating the survival advantage (Figure 7C). The depletion of CD4^+^ T cells seemed to also have a profound effect on survival, reducing the median survival to 31 days. Interestingly, we also observed a decrease in the survival of mice treated with clodrolip (clodronate-liposomes), indicating that CAR expressing macrophages may indeed play a role in tumor eradication in this model. Previous studies have indicated the eradication of intraperitoneal RMA tumor challenges are facilitated through the combined efforts of CD8^+^ T cells and NK cells [34]. As the depletion of either CD8 or NK cells completely abrogated the survival advantage observed in immunocompetent mice, we explored whether the depletion of both CD8^+^ T cells and NK cells would further reduce the survival time in F38 *vav*-CAR mice. Indeed, we observed a reduction in the median survival time of mice treated with the combination depletion (NK cells and CD8^+^ T cells) compared to those treated with the single depletion (24 days for combination, 27 days for NK, 35 for CD8).

Taken together, the data indicates that the presence of NK cells and macrophages as well as both CD4^+^ and CD8^+^ T cells are integral in the inhibition of Her2^+^ tumors in the *vav-*CAR model.

## DISCUSSION

Despite the recent success using adoptively transferred CAR-expressing CTLs in hematological cancers, further work is required in order to gain similar levels of success in other cancer types. There is mounting evidence demonstrating the role for other immune cell types in adoptive immunotherapy, with numerous studies reporting enhanced anti-tumor effects with the co-transfer of unmodified or genetically modified CTLs in addition to other helper immune subsets [11–13, 15]. However, due to current limitations in genetic modification techniques, the role and function of other immune subsets when expressing a CAR have yet to be fully explored. We developed a novel mouse model utilizing a pan-hematopoietic promoter to drive the expression of a CAR directed against the tumor antigen ErbB2 within most immune subsets. Using this model, we were able to demonstrate the ability of the *vav*-promoter in driving CAR expression throughout multiple immune lineages and subsets.

The curative potential of CAR expressing T cells has been demonstrated and well characterized in both mouse models and in clinical trials [35]. Despite similar methods of activation and similar ratios of CD4 to CD8 T cells, we observed almost a ten-fold difference in TNF-α, IL-6 and IFN-γ released from F9 T cells compared to F38 T cells in response to tumor cells 24JKERB. Whole genome analysis previously revealed F9 harboured almost 270 copies of the *vav-*CAR transgene, while F38 only had 7 copies [29]. This difference in gene dosage and the number of receptors present would have likely increased the strength of the interaction between the CAR and its target and subsequently, the strength of the downstream signal. This may have thus impacted the function of these cells, and whether they were indeed primed for cytotoxicity or cytokine secretion [36]. Therefore, we hypothesize that this may have accounted for the difference observed in cytokine secretion between the two founders. Indeed while CAR T cells from both strains of *vav-*CAR mice were able to mediate antigen specific cytotoxicity against the tumor line, 24JK, a higher level of background killing (antigen independent) was observed from F9 mice, indicating potentially a difference in the activation levels and baseline cytotoxicity of these T cells.

The use of CAR expressing murine NK cells in adoptive immunotherapy has not been extensively studied. Previous work from our lab has demonstrated the potential for primary murine NK cells genetically modified with a CAR to exhibit antigen specific anti-tumor function [22]. In the current study, when comparisons between the level of cytotoxicity against RMAERB from WT and CAR NKs were drawn, we observed a significantly higher level of killing from CAR NKs compared to WT NKs at all four effector to target ratios, however we also observed a higher level of background killing from CAR NKs against the RMA targets. Therefore, the genetic modification of NK cells with a CAR has been shown to increase their anti-tumor potential but at the price of a higher basal level of cytotoxicity. Careful consideration must be taken to ensure the cytotoxic functions are restricted to antigen specific targets in future clinical trials.

Given the difficulties in isolating and expanding numerous subsets to determine their anti-tumor function, we decided to approach this in a different manner by challenging *vav*-CAR mice intraperitoneally with the RMAERB cell line, and observe whether the absence of specific immune subsets would increase or decrease the survival outcome. The differences in survival between F9 and F38 mice when challenged with RMAERB cells indicated the necessity for lymphocytes, in particular CD8^+^ T cells for long-term survival.

Surprisingly, we observed a significant reduction in the survival of macrophage depleted (clodrolip treated) mice, in the F38 strain, with a reduction in median survival time from 100 to 43 days, suggesting a role for macrophages in tumor inhibition. There are numerous functions that macrophages could play to contribute to tumor rejection in this model; including phagocytosis, antigen presentation or cytokine secretion to activate other effector cells. However, the scope of this study did not extend as far to determine their exact role in this tumor model. While our *in vitro* and *in vivo* data indicates a potential role for CAR expressing macrophages in adoptive immunotherapy, we cannot rule out the complexity of the immune compartment in the peritoneum. The introduction of tumor cells in the peritoneum would result in inflammation at the injection site, and subsequently attract various immune responders. As clodrolip does not specifically target macrophages, but rather phagocytic cells, it is likely that clodrolip treatment in our experiment depleted numerous phagocytic subsets. A macrophage specific depletion, such as crossing the vav-CAR mice with CD11b-DTR transgenic mice [37] may be useful to determine the exact contributions of CAR-macrophages in this model. In addition, the polarisation and phenotype of tumor-infiltrating macrophages, be they M1 or M2, can greatly increase or decrease the anti-tumor efficacy and the function of these cells, respectively [38, 39]. Future studies using this model may benefit from specifically depleting tumor-promoting macrophages (M2) or polarising them into a more anti-tumor phenotype (M1). Overall, this data suggest that a coordinated response is required from multiple arms of the immune system for efficient anti-tumor function, and indicates a potential role for CAR expressing macrophages in adoptive immunotherapy.

In our studies into the contributions of cell types to antitumor activity, we used a depleting antibody specific for CD8-alpha (to deplete T cells) and clodrolip (to deplete macrophages). However, since CD8-alpha is also expressed on a proportion of dendritic cells (DCs) and clodrolip also depletes some DCs, we cannot rule out a contribution from DCs in the effect. It would be informative in future experiments to use more specific reagents, for example using an anti CD8-beta antibody that does not deplete DCs. Strikingly, the depletion of CD8^+^ T cells had a less dramatic effect on the survival of F38 mice compared to the depletion of NK cells or CD4^+^ T cells. As the percentage of NK cells and the subsequent proportion of CAR^+^ NK cells is similar between both F9 and F38 mice (F9 1.96 ± 0.22 total NKs, 56.71% CAR^+^, F38 3.08 ± 0.15 total NKs, 44.93% CAR^+^, mean ± SEM, proportion of CAR^+^ NKs in total population, [29]), and we observed significant differences in survival times in the RMAERB challenge (Figure 6), this indicates that the NK cells themselves may not be playing a direct cytotoxic role in this tumor model. Rather, the reduction in CAR^+^ CD8^+^ T cell population in the F9 model suggests that CD8^+^ T cells are integral for tumor rejection, and perhaps the presence of CAR^+^ CD4^+^ T cells or CAR^+^ NKs are required to prime and coordinate the overall immune response, providing sufficient activation either through direct contact or via cytokine secretion to facilitate the actions of CAR^+^ CD8^+^ T cells. As the immune system functions on a finite balance, one could postulate the depletion of an integral anti-tumor effector such as a CD8^+^ T cell, CD4^+^ T cell or NK cell may be sufficient to offset this balance, and thus have a greater effect on the activity of the remaining immune system than just the absence of the cell itself.

In line with our hypothesis, the depletion of both NK cells and CD8^+^ T cells together had the greatest effect in reducing the survival time in this tumor model (combo 24 days, α-CD4 31 days, α-CD8 35 days, Asialo 27 days, Clodrolip 43 days, IgG2a control > 100 days), suggesting that a synergistic effect may be occurring. However further investigation to delineate the exact role for CAR NKs and CAR T cells are required. The *vav-*CAR model provides the perfect platform to enable testing of such combinations, allowing for the isolation of stably expressing CAR NK cells and CAR T cells without the requirement for genetic modification. Future experiments with this model will aim to dissect the potential of these two immune cells and in particular, whether adoptive transfer of CAR T cells and NK cells can synergise and what factors are required for enhancing CAR^+^ CD8^+^ T cell function.

One caveat of CAR T cell therapy is the “on-target/off-tumor” effect and the subsequent side effects and toxicity that ensue [35, 40]. As CAR-modified NK cell lines have proven to be safe and are able to mediate cytotoxicity in a highly antigen dependent manner [25, 41, 42], it may be of interest to combine primary CAR modified T cells with CAR modified NK-92 cells in future studies. Not only would this reduce the time required for *ex vivo* expansion of the primary CAR T cells (thus enabling the use of “younger” T cells [43–45]) but a reduction in the number of T cells transferred may also correlate in a reduction in “on-target/off-tumor” effects [4, 46]. Further pre-clinical analysis must be performed to determine the anti-tumor potential of the combination of these two subsets. The use of genetically modified IL-15 secreting NK-92 cells or IL-2/IL-15 independent NK-92 cell lines may be used in these studies to alleviate any potential competition for cytokines between the two cell subsets [47–49]. In addition, the genetic modification of this cell line to secrete cytokines or factors, which enhance T cell survival and proliferation may also reduce the need to deliver high doses of exogenous cytokines alongside the adoptive transfer.

There have been recent studies highlighting the potential to genetically modify hematopoietic stem cells (HSCs) with CARs to allow for a long term and continuous source of CAR expressing immune cells [50]. The many advantages of CAR modified HSCs include the potential for a younger, more naive population of CAR expressing T cells with a greater chance of developing into long term memory cells. In addition, as the generation of NK cells and myeloid cells with CARs would be relatively faster than the development of the adaptive immune system, this may provide early protection against residual tumors during the time taken for thymopoiesis of CAR bearing T cells in the stem cell setting [17, 51]. The feasibility of moving this type of therapy to the clinic (after vigorous pre-clinical studies) would be relatively straight forward, where CAR modified HSCs would be transplanted after conditioning treatments, such as in a normal bone marrow transplant [50]. Several mouse models, using TCR, CAR or ‘universal receptor’ (UR) modified HSCs have been developed in order to study the function of genetically modified myeloid cells and other non-T lymphocytes [16, 17, 50–53]. The development of the *vav*-CAR mouse model may contribute greatly to this field of adoptive immunotherapy. We have demonstrated in our previous studies the expression of the anti-Her2 CAR is present on multiple immune subsets, including those of myeloid origin. The use of *vav-*CAR HSCs from transgenic mice would eliminate the laborious task of genetically modifying HSCs with CARs, and furthermore, ensure stable and consistent transgene dosages between experiments.

The *vav*-CAR mouse model is a novel and unique tool to further study the anti-tumor potential of a range of immune subsets when genetically modified to express a CAR. We have previously demonstrated the ability of the *vav* promoter to drive the expression of a CAR throughout multiple immune subsets [29]. In this study, we demonstrate the anti-tumor function of multiple immune subsets from these mice. *Vav*-CAR mice challenged with Her2 expressing tumors were able to mount an effective anti-tumor immune response, resulting in tumor eradication and long term survival, and this was shown to be dependent on both NK cells and CD8^+^ T cells with an important role also shown for CD4^+^ T cells and macrophages. In future studies the *vav*-CAR mouse will enable testing the anti-tumor function of various adoptively transferred immune cell combinations. It remains to be established whether all effectors in these combinations require the ability to recognise the tumor antigen or if recognition by a key player, such as a CTL, may be sufficient to activate and coordinate with other unmodified effector cells. Elucidating the mechanisms between different genetically modified immune subsets may lead to significantly improving the therapeutic application of adoptive immunotherapy, particular in the solid cancer setting.

## MATERIALS AND METHODS

### Cell culture and mouse models

The murine 24JK fibrosarcoma cell line [54] was kindly donated by Dr. Patrick Hwu (NIH, Bethesda, MD) and maintained at 37°C in 5% CO_2_ in RPMI-1640 media supplemented with 5% heat-inactivated fetal calf serum (FCS) with 2 mmol/L glutamine, 1 mmol/L sodium pyruvate, 0.1 mmol/L nonessential amino acids, 100 U/mL penicillin and 100 μg/mL streptomycin (Life Technologies). The murine breast adenocarcinoma cell line E0771 (LMC variant) was kindly donated by Professor Robin Anderson (Peter MacCallum Cancer Centre, Victoria, Australia) and maintained in RPMI with supplements as above. The murine RMA T cell lymphoma cell line was derived from the Rauscher murine leukemia virus-induced RBL-5 cell line and maintained at 37°C in 10% CO_2_ in Dulbecco's modified Eagle medium (DMEM) supplemented as above [55]. All cell lines were previously retrovirally transduced with a retroviral vector encoding the cDNA for human Her2 [21]. *Vav-*CAR mice were generated and characterized as previously described [29]. Heterozygous mice were used unless stated. C57BL/6-Her2 mice express human Her2 under the mouse WAP promoter [56, 57]. Mice were bred and maintained under specific pathogen-free conditions within the animal experimentation facility at the Peter MacCallum Cancer Centre. All mice experiments were performed following the Peter MacCallum Cancer Centre Animal Experimentation Ethics Committee guidelines.

### Flow cytometry

Spleens from WT and *vav-*CAR mice were dissociated in phosphate-buffered saline (PBS) and processed into single cell suspensions through a 70 μm filter. Splenocytes were treated with ammonium chloride potassium (ACK) lysis buffer to remove red blood cells. 5 × 10^5^ splenocytes, *in vitro* activated cells and peritoneal exudate cells (PECs) were resuspended in Fc receptor-blocking buffer (anti-mouse FcγRII/FcγRIII, clone 2.4G2 made in house from tissue culture supernatant) for 10 minutes prior to staining with the following antibodies; TCRβ-PerCP-Cy5.5 (clone H57-597), CD3-e450 (clone 17A2), CD4-APC-eF780 (clone RM4-5), CD8-Pe-Cy7 (clone 53-6.7), CD11B-APC (clone M1/70), F4/80-Pe-Cy7 (clone BM8), NK1.1-Pe-Cy7 (clone PK136), CD49b-APC (clone DX5) (all from eBioscience, California, USA), α-c-myc-tag Alexa488 (clone 9B11) (Cell Signaling, Massachusetts, USA) or mouse immunoglobulin isotype IgG2a Alexa488 (Invitrogen, Scoresby, Australia).

### T cell activation

Splenocytes were dissociated into single cell suspensions and treated with ACK lysis buffer as above. Remaining leukocytes were resuspended at 2.5 × 10^7^ cells/well in 5 ml of RPMI with additives (see cell culture methods section) and were activated overnight with α-CD3ε (clone 145-2C11) (500 ng/ml) and α-CD28 (clone 37.51) (500 ng/ml) (BD Bioscience) with recombinant human IL-2 (rhIL-2) (100 U/ml) and mouse IL-7 (mIL-7) (2 ng/ml) in 5% CO_2_ at 37°C. The cells were harvested the next day and centrifuged at 400 g for 4 minutes to remove activating antibodies. Cells were then resuspended in RPMI with additives including rhIL-2 (100 U/ml) and mIL-7 (2 ng/ml). Functional assays and immunophenotyping were performed on days 5 to 7 post activation.

### NK isolation and expansion

Spleens from WT and homozygous F38 mice were processed as above to produce single cell suspensions. No ACK lysis was performed. 5 × 10^7^ splenocytes were resuspended in 500 μl of Facs buffer (PBS + 2% FCS). NK cells were isolated by negative selection using the StemCell EasySep Mouse NK Cell Enrichment kit (StemCell) according to manufacturer's instructions. NK cells (7 × 10^5^) were resuspended in 1 ml of RPMI plus additives to a total of 20% FCS in addition to 1000 IU/ml of rhIL-2 and 50 mM β-Mercaptoethanol (BME). On day 5 post isolation, 500 μl of the NK culture was transferred to a new well and refreshed with RPMI plus additives in addition to BME and 100 IU/ml of rhIL-2. Functional assays were performed on day 7 post isolation.

### Cytometric bead array

For T cells; 2 × 10^6^ *in vitro* activated T cells were cultured overnight with the following conditions; media alone, immobilized α-CD3ε and α-CD28 (50 ng/ml), immobilized α-IgG2a κ (BD Bioscience), immobilized α-c-myc tag (clone 9B11) (Cell signaling) or 1 × 10^6^ tumor targets 24JK or 24JKERB. For macrophages; naïve peritoneal exudate cells (PECs) from WT and *vav*-CAR mice were harvested in the absence of thioglycollate stimulation. 1 × 10^5^ PECs were co-cultured overnight with media (DMEM) containing either lipopolysaccharide (LPS) at (5 μg/ml), or immobilized α-c-myc tag or IgG2a at (0.5 μg/ml). For NKs; 1.25 × 10^5^ *in vitro* activated NK cells were co-cultured with media alone, or with positive stimulation via phorbol 12-myristate 13-acetate (PMA) and ionomycin (50 ng/μl and 1 μg/ml respectively), or immobilized α-IgG2a κ, or immobilized α-c-myc tag or tumor targets 24JK/24JKERB or RMA/RMAERB at a 1:1 ratio (1.25 × 10^5^ cells of each). Supernatant was harvested the next day and analysed by cytometric bead array (CBA) for the following cytokines and chemokines; IL-4, IL-2, IL-17A, TNF-α, IL-6 and IFN-γ for T cells and IL-17A, TNF-α and IFN-γ for NK cells using the Facs Verse (BD Bioscience). Results were analysed using the FCAP Array Software (BD Bioscience).

### Phagocytosis assay

Methodology to detect macrophage phagocytosis was adapted from [58]. WT and *vav*-CAR mice were injected with 1ml of thioglycollate intraperitoneally. PECs were harvested on day 4 by intraperitoneal wash. Tumor cells (2.5 × 10^5^ RMA/ERB) were labeled with carboxyfluorescein succinimidyl ester (CFSE) and co-cultured with 2.5 × 10^5^PECs for 1 hour at 37°C in 10% CO_2_ in a 24 well plate. After 1 hour, the plate was centrifuged at 400 g for 4 minutes. The cells were incubated with Fc receptor block for 10 minutes, stained with F4/80-Pe-Cy7 (clone BM8) and CD11B-APC (clone M1/70) (eBioscience) and analysed by flow cytometry. Macrophages were gated on as being F4/80^+^and CD11b^+^ and the proportion and mean fluorescence intensity (MFI) of CFSE positive macrophages was assessed as a measure of phagocytosis.

### Chromium release assay

Tumor targets 24JK and 24JKERB or RMA and RMAERB were labeled with ^51^Cr and plated at either 5 × 10^3^ or 2 × 10^4^ cells/well in a 96-well U-bottom plate. Activated effector cells were co-incubated at varying effector to target ratios (1:1, 5:1, 10:1, 20:1, 40:1) and incubated for 4 hours at 37°C in 5% CO_2_. Specific chromium release was determined by the following equation;

(% chromium release - % spontaneous chromium release) × 100

(% total chromium release - % spontaneous chromium release)

with total chromium release determined by the addition of sodium dodecyl sulphate (SDS). The level of chromium release was determined using a Wallac Wizard 1470 automatic gamma counter (Amersham).

### *In vivo* adoptive transfer

Human Her2^+^ transgenic mice were injected subcutaneously (SC) with 1 × 10^5^ E0771ERB cells [56]. The mice were then sub-lethally irradiated with 5 Gy irradiation 7 days later and treated with 3–5 × 10^6^ T cells intravenously on day 7, 8 and 14. Exogenous IL-2 (50,000 IU/200 μl) was administered intraperitoneally on day 7 and thereafter twice a day for four days post treatment (9 doses total). Tumors were measured twice a week and mice were euthanized when tumors exceeded the ethical limit of 150 mm^2^. For NK and T cell adoptive transfer, C57BL/6 mice were injected intraperitoneally with 1 × 10^6^ RMAERB cells and then treated on the same day with 5 × 10^6^ effector cells intraperitoneally. Non-treated mice served as controls. Mice were euthanized when showing signs of illness or when abdomen diameter had increased over 30%.

### RMA challenge and *in vivo* depletion studies

RMA or RMAERB cells were washed twice with PBS and resuspended at 5 × 10^6^ cells/ml. Naïve C57BL/6 mice or homozygous *vav*-CAR mice were injected with 1 × 10^6^ tumor cells (200 μl) using a 26G needle into the peritoneum and monitored for signs of illness and survival. Mice were euthanized when showing signs of illness or when the abdomen diameter had increased over 30%. For depletion studies, mice received primary depletion 2 days prior and on the day of tumor inoculation using the following antibodies; Asialo GM1 (100 μl/mouse) (Wako), α-CD8 (clone YTS169.4) at 300 μg/mouse, clodronate liposomes (Clodrolip) at 250 μg/mouse, α-CD4 (GK1.5) at 300 μg/mouse, or an irrelevant antibody rat IgG2a (clone 2A3) at 300 mg/mouse (clone 2A3). Weekly maintenance depletions were administered with the following concentrations; Asialo GM1 at 100 μl/mouse,α-CD8 at 100 μg/mouse, Clodrolip at 250 μg/mouse, α-CD4 at 100 μg/mouse or rat IgG2a irrelevant antibody at 100 μg/mouse for the first 6–7 weeks post tumor inoculation. Mice treated with the combination received both α-CD8 and Asialo GM1 at the same concentration as the single treatments. Surviving mice were rechallenged with 1 × 10^6^ RMAERB cells injected into the peritoneum at day 60 and monitored for survival.

### Statistics

Statistical significance was analysed using an unpaired Student's *T* test. Tumor growth measurements in Figure 5 were analysed using a two-way ANOVA.
